# Multiple interactions between the alpha_2C_- and beta_1_-adrenergic receptors influence heart failure survival

**DOI:** 10.1186/1471-2350-9-93

**Published:** 2008-10-23

**Authors:** Sharon LR Kardia, Reagan J Kelly, Mehdi A Keddache, Bruce J Aronow, Gregory A Grabowski, Harvey S Hahn, Karen L Case, Lynne E Wagoner, Gerald W Dorn, Stephen B Liggett

**Affiliations:** 1 Department of Epidemiology, School of Public Health, University of Michigan, 109 Observatory St., Ann Arbor, MI 48109-2029 USA; 2 Cincinnati Children's Hospital Medical Center, Cincinnati, OH USA; 3 Department of Internal Medicine, University of Cincinnati, Cincinnati, OH USA; 4 Center for Pharmacogenomics, Washington University School of Medicine, St Louis, MO 63110 USA; 5 Department of Medicine, Cardiopulmonary Genomics Program, University of Maryland, 20 Penn St., HSF-II, Baltimore, MD 21201-1075 USA

## Abstract

**Background:**

Persistent stimulation of cardiac β_1_-adrenergic receptors by endogenous norepinephrine promotes heart failure progression. Polymorphisms of this gene are known to alter receptor function or expression, as are polymorphisms of the α_2C_-adrenergic receptor, which regulates norepinephrine release from cardiac presynaptic nerves. The purpose of this study was to investigate possible synergistic effects of polymorphisms of these two intronless genes (*ADRB1 *and *ADRA2C*, respectively) on the risk of death/transplant in heart failure patients.

**Methods:**

Sixteen sequence variations in *ADRA2C *and 17 sequence variations in *ADRB1 *were genotyped in a longitudinal study of 655 white heart failure patients. Eleven sequence variations in each gene were polymorphic in the heart failure cohort. Cox proportional hazards modeling was used to identify polymorphisms and potential intra- or intergenic interactions that influenced risk of death or cardiac transplant. A leave-one-out cross-validation method was utilized for internal validation.

**Results:**

Three polymorphisms in *ADRA2C *and five polymorphisms in *ADRB1 *were involved in eight cross-validated epistatic interactions identifying several two-locus genotype classes with significant relative risks ranging from 3.02 to 9.23. There was no evidence of intragenic epistasis. Combining high risk genotype classes across epistatic pairs to take into account linkage disequilibrium, the relative risk of death or transplant was 3.35 (1.82, 6.18) relative to all other genotype classes.

**Conclusion:**

Multiple polymorphisms act synergistically between the *ADRA2C *and *ADRB1 *genes to increase risk of death or cardiac transplant in heart failure patients.

## Background

Congestive heart failure can be caused by a wide array of myocardial insults. Although this etiological heterogeneity has yet to be well characterized, the role of neurohormonal pathways in the progression of heart failure is well established [1-3]. Prejunctional α_2_-adrenergic receptors (α_2A _and α_2C_) regulate the release of norepinephrine from cardiac sympathetic nerves in a negative feedback manner. When these receptors are ablated in mouse models, uncontrolled norepinephrine release causes a lethal cardiomyopathy [4,5]. Moreover, the released norepinephrine activates β_1_-adrenergic receptors (β_1_AR) expressed on cardiac myocytes which are coupled to stimulatory G-proteins that propagate signals to downstream effectors such as adenylyl cyclase, ion channels, and phospholipases [6]. Prolonged activation of cardiomyocyte β_1_AR signaling by any number of pharmacologic or genetic means typically results in hypertrophy, ventricular dysfunction, ventricular remodeling, or frank failure [7]. Thus, sympathetic activation of the heart is coordinated in part by these two adrenergic receptors, in which genetic variation of expression or function could act to modify the progression of heart failure. And indeed, substantial variation in the progression, mortality, and treatment response of heart failure between otherwise similar individuals is well recognized [8-10].

Previous studies of a common single nucleotide polymorphism C->G in the β_1_AR gene (*ADRB1*) at codon 389, which results in an arginine for glycine substitution (Arg389Gly), have shown enhanced coupling of the Arg variant to G_s _in recombinant cells [11]. Arg389 is associated with differential exercise capacity in heart failure patients [12], response to beta-blockers [13,14], hypertension [15], and risk of myocardial infarction [16]. In transgenic mice, the Arg variant has been associated with early enhanced cardiac function but in older mice a predisposition to heart failure [17].

A common insertion/deletion polymorphism in African-Americans resulting in a consecutive four amino acid loss (322–325) in the α_2C_AR gene (*ADRA2C*) has been found to reduce receptor function [18], leading to a loss of normal synaptic autoinhibitory feedback and concomitant enhanced release of norepinephrine [19]. Given the potential synergistic effects of these polymorphisms in the *ADRB1 *and *ADRA2C *genes, two previous studies have investigated and identified epistatic interactions affecting heart failure risk [20] and response to β-blockers in heart failure patients [21]. In both studies, only two putative functional polymorphisms (i.e., *ADRB1 *Arg389Gly and *ADRA2C *ins/del 322) were examined. Multiple additional polymorphisms in both of these two intronless genes have been recently identified and characterized in whole-gene transfection studies, some of which alter protein expression [22,23]. Here we examine whether there is evidence for more complex intragenic and intergenic epistatic effects on heart failure phenotypes and survival in these genes utilizing 16 DNA sequence variations in the *ADRA2C *and 17 DNA sequence variations in the *ADRB1 *genes.

Intergenic epistasis, or interaction between two genes, occurs when the phenotypic effects of a variation in one gene is affected by a variation in a second gene. This could result from conformational changes that prevent physical interaction between the two gene products, or from a change in the ability of one gene to regulate the expression of the other or, the pathologic pathway involves both genes in a manner that their downstream effects converge to alter critical events. Intragenic epistasis occurs when a variation in one location of a gene influences the phenotype differently depending on other variations within the same gene. This type of epistasis is most notable when two different amino acid substitutions within a gene result in differentially functioning the resulting protein. However, it is also seen to have an effect on gene expression and processing.

## Methods

### Study Population

The 655 Caucasian heart failure patients were identified and enrolled in the study at the University of Cincinnati, Cincinnati, OH, between 1999 and 2004. The study was restricted to Caucasians because of the significant differences in the allele frequencies between those of African- and European-descent in these two genes [22,23], and a smaller number of potential black enrollees. Other enrollment criteria were: age of 18 to 80 years, left ventricular ejection fraction (LVEF) of less than 40%, and New York Heart Association heart failure class II-IV. The primary study endpoint was the combined event of death or cardiac transplantation. Descriptive statistics of this cohort are given in Table 1. The human study protocols were approved by the institutional review board of the University of Cincinnati, and subjects provided written informed consent.

**Table 1:** Descriptive statistics for the heart failure cohort

**Variable**	**N**	**Mean ± SD**
Age at onset of heart failure (yrs)	655	53.79 ± 12.73
Follow-up time (yrs)	655	3.16 ± 2.70
Height (cm)	554	172.2 ± 10.06
Weight (kg)	560	85.82 ± 20.73
Left Ventricular Ejection Fraction	415	27.72 ± 13.72
Left Ventricular Mass indexed to Body Surface Area	456	189.14 ± 69.04
Fractional Shortening	492	21.97 ± 11.16
**Variable**	**N**	**%**
		

Males	453	69.2
Hypertension	310	47.9
Beta Blocker Use	455	69.6
ACE Inhibitor Use	550	84.0
Had Endpoint:		
Death	127	19.4
Cardiac Transplant	171	26.1
Heart Failure Etiology:		
Dilated Cardiomyopathy	382	58.3
Ischemic Cardiomyopathy	259	39.5
Other	14	2.1

### Genotyping

Sequence variants have been previously identified for *ADRA2C *[22] and *ADRB1 *[23] and the nomenclature used in these papers is retained in the current work to maintain consistency. Both genes are intronless, and the A of the initiation codon is denoted as +1, proceeding in the positive direction 5' -3' through the coding and 3' UTR. In the 5' -flanking region the most 3' non-coding nucleotide prior to the ATG is denoted as -1 and the numbering proceeds in the negative direction. The genotyping was performed on the variant sites shown in Table 2. For orientation purposes +1 of *ADRA2C *is nucleotide 2638 of AY605898, and +1 of *ADRB1 *is nucleotide 36085 of AL355543. The variants are deposited in the PharmGKB (*ADRA2C*) and SeattleSNPs (*ADRB1*) public databases ( and , respectively). Genotyping was performed on genomic DNA derived from blood samples, by sequencing PCR products spanning one or two variant positions, using an ABI 3730 sequencer. Variants detected by alignment to a reference were verified by visual electropherogram examination.

**Table 2:** Summary of the SNP frequency distributions

**SNP**	**Genotype (N)**	**Minor Allele**	**Minor Allele Freq**	**H-W P-value**
*ADRA2C*(-2579 T/C)	568	C	0.013	0.000
*ADRA2C*(-2416 C/G)	613	G	0.001	1.000
*ADRA2C*(-2357 C/T)	615	T	0.001	1.000
*ADRA2C*(-2280 G/T)	612	T	0.002	1.000
*ADRA2C*(-2069 C/T)	602	T	0.111	0.151
*ADRA2C*(-1926 G/A)	608	A	0.031	1.000
*ADRA2C*(-1692 T/G)	624	G	0.069	0.066
*ADRA2C*(-1513 T/G)	626	G	0.018	1.000
*ADRA2C*(-965 G/C)	634	C	0.001	1.000
*ADRA2C*(-940 G/A)	634	A	0.136	0.615
*ADRA2C*(-933 C/A)	634	A	0.028	1.000
*ADRA2C*(-696 C/G)	426	G	0.058	0.640
*ADRA2C*(-241 C/G)	644	G	0.001	1.000
*ADRA2C*(-230 T/C)	644	C	0.019	1.000
*ADRA2C*(+964 ins/del)	587	del	0.066	0.010
*ADRA2C*(+1736 G/C)	651	C	0.235	0.387
*ADRB1*(-4415 T/C)	572	C	0.287	0.477
*ADRB1*(-4267 ins/del)	573	ins	0.001	1.000
*ADRB1*(-3641 C/T)	593	T	0.146	0.073
*ADRB1*(-3598 C/T)	593	T	0.066	1.000
*ADRB1*(-3255 A/C)	608	C	0.141	0.243
*ADRB1*(-2915 G/A)	643	A	0.001	1.000
*ADRB1*(-2853 G/A)	644	A	0.001	1.000
*ADRB1*(-2827 C/A)	644	A	0.030	0.447
*ADRB1*(-2639 T/C)	637	C	0.433	0.568
*ADRB1*(-2297 T/G)	607	G	0.151	0.082
*ADRB1*(-2142 T/C)	608	C	0.146	0.250
*ADRB1*(-1294 G/A)	579	A	0.003	1.000
*ADRB1*(-1121 T/C)	580	C	0.003	1.000
*ADRB1*(-517 T/C)	627	C	0.131	0.015
*ADRB1*(+145 A/G)	584	G	0.146	0.018
*ADRB1*(+315 G/T)	587	T	0.002	1.000
*ADRB1*(+1165 G/C)	557	G	0.268	0.665

### Statistical Methods

Allele frequencies were estimated using standard gene counting methods. Hardy-Weinberg disequilibrium was tested using Weir's method [24]. Linkage disequilibrium was assessed using the r^2 ^statistic [24]. Cox Proportional Hazards modeling [25]. was used to test for significant effects for each polymorphism (separately) and pairwise epistatic effects after adjustment for age at initial diagnosis, β-blocker usage, hypertension status, and sex. Genotypes for each polymorphism were coded using two dummy variables where the most frequent genotype was considered the reference group. Epistasis was assessed by including interaction terms into the Cox models.

To adjust for multiple testing we used Storey's modification of the false discovery rate (FDR) method of Benjamini and Hochberg [26] that takes into account the correlation among tests due to linkage disequilibrium. We used an FDR cutpoint of 0.30. To assess whether the information from the Cox proportional hazards modeling of genetic effects provides a useful prediction of a new patient's risk of death or cardiac transplant, we implemented a leave-one-out cross-validation approach [27]. Each individual was sequentially left out and a Cox proportional hazards model for time from study enrollment to death or cardiac transplant was estimated. Using the coefficients estimated with the *n*-1 individuals, a survival risk was calculated for the individual left out. These risks were then used as the predictor in a new Cox proportional hazards model. Because each individual was omitted from the model used to calculate their survival risk, the performance of a model using these risks as predictors approximates the predictive ability of the association in an independent sample drawn from the same population.

In order to assess the overall impact of high-risk two-locus genotype classes on risk of death or cardiac transplant, a pooling procedure was performed. If an individual possessed one or more two-locus genotype class that had a relative risk whose 95% confidence interval was greater than 1.0, they were assigned the label "has high-risk genotype." All other individuals were assigned the label "does not have high-risk genotype." Cox proportional hazards modeling was then performed with this label as the explanatory variable.

## Results

**Figure 1:**
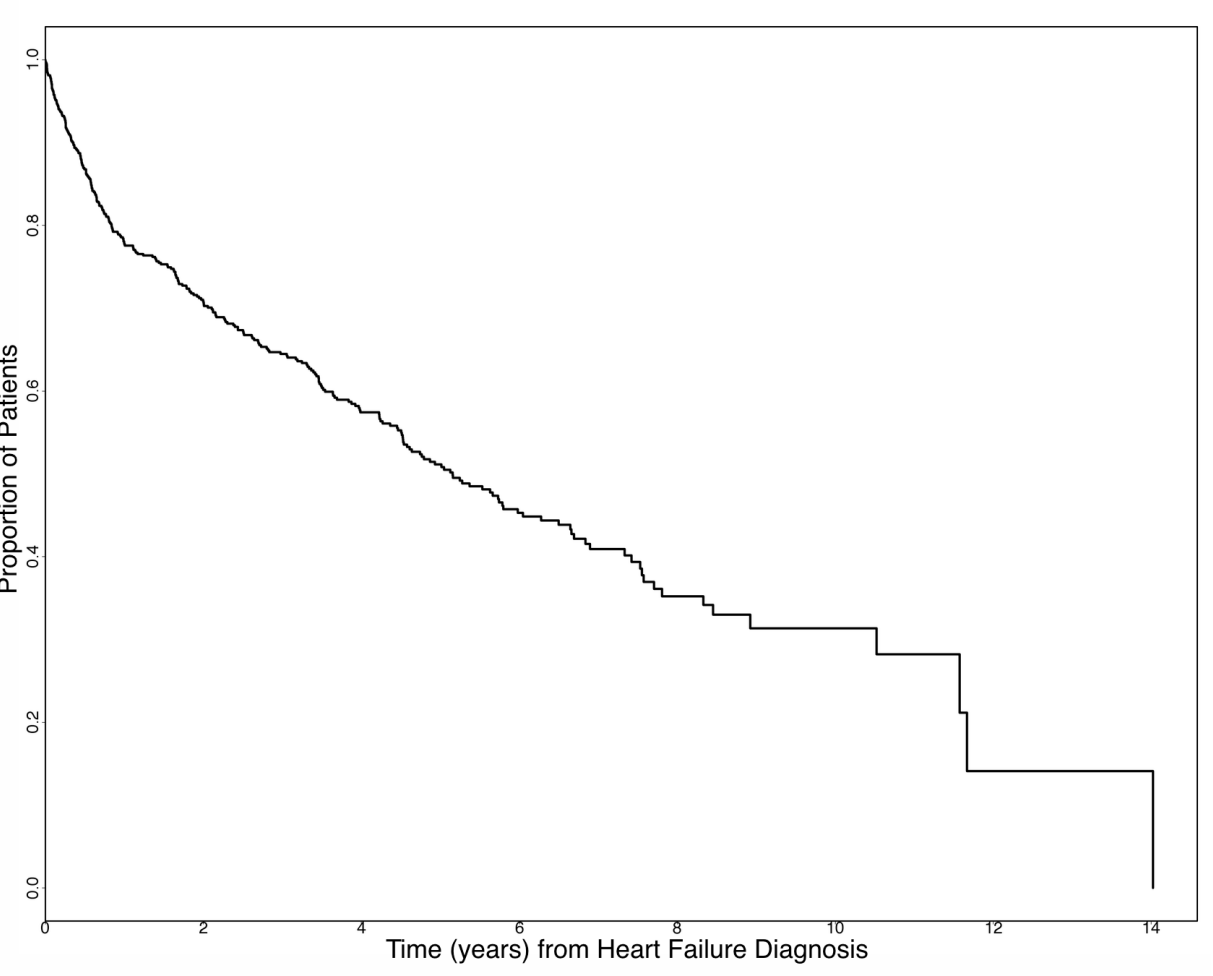
Kaplan-Meier curve of time from heart failure diagnosis to death or cardiac transplant.

**Figure 2:**
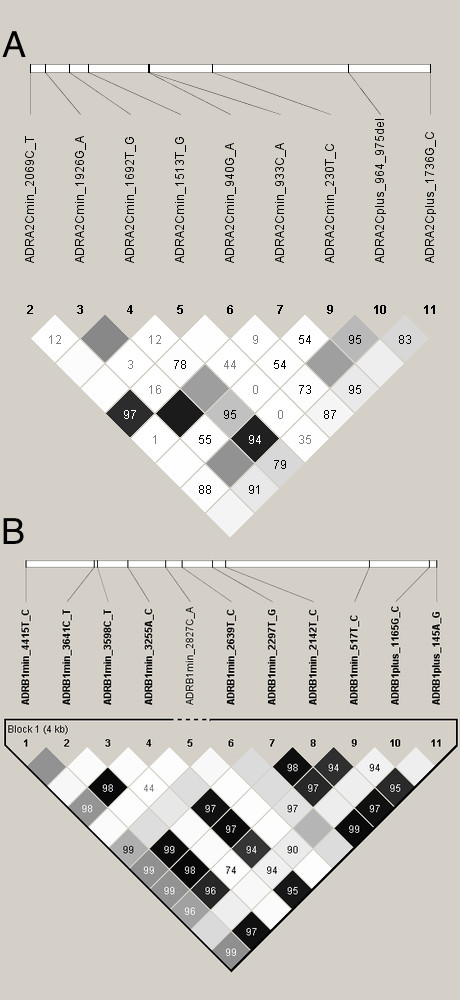
A. Linkage disequilibrium among *ADRA2C *polymorphisms B. Linkage disequilibrium among *ADRB1 *polymorphisms.

The 655 subject heart failure cohort was 69.2% male, with 47.9% having a history of hypertension, 84.0% receiving ACE inhibitor therapy and 69.6% receiving β-blocker therapy (Table 1). Ischemic cardiomyopathies made up 39.5% of the cohort, idiopathic dilated cariomyopaties comprised 58.3% of the cohort, and the remaining 2.1% of the cohort had other causes of heart failure, such as primary valvular defects. They had an average age of heart failure onset of 53.8 years and an average follow-up time of 3.16 years. Figure 1 shows a Kaplan-Meier curve of time from heart failure diagnosis to death or cardiac transplant for this cohort. The minor alleles and the frequencies of the 16 DNA sequence variations in the *ADRA2C *and 17 DNA sequence variations in the *ADRB1 *genes are displayed in Table 2. Eleven of the 16 variants genotyped in the *ADRA2C *gene and 11 of the 17 in the *ADRB1 *gene had frequencies greater than 1% and are considered polymorphic. Four polymorphisms, *ADRA2C*(-2579 T/C), *ADRA2C*(+964/965 ins/del), *ADRB1*(-517 T/C), and *ADRB1*(+145 A/G), were significantly out of Hardy Weinberg equilibrium (HWE) (P < 0.05). The linkage disequilibrium between polymorphisms is illustrated in Figure 2 and indicates that several polymorphisms within each gene have significant frequency correlations.

Given the large number of tests for main effects and epistatic effects using these 22 polymorphic loci we used both the false discovery rate and cross-validation methods to reduce the probability of false positive results. Table 3 provides a summary of the tests of association between the polymorphism and age of onset, LVEF, LV mass, and survival. Overall, only two polymorphisms had significant main effects on the survival distribution and 8 pairs of polymorphisms had epistatic effects on survival that were significant (after adjustment by FDR) and cross-validated.

**Table 3:** Summary of the significant, false discovery rate adjusted, and cross-validated ADRA2C and ADRB1 polymorphism effects and interactions on age of onset, LVmass, LVEF, and heart failure survival

ED (N = 655)								
	Age of Onset		LVMass Adj*		LVEF Adj**		Survival	
	
	SNP Main Effects	SNP-SNP Interaction	SNP Main Effects	SNP_SNP Interaction	SNP Main Effects	SNP_SNP Interaction	SNP Main Effects	SNP_SNP Interaction
	
Number of tests	22	209	22	200	22	204	22	231
P < 0.10	2	24	1	8	2	16	4	50
FDR (<0.30)	0	0	0	0	2	0	4	44
Cross Validation***	0	0	0	0	1	0	3	10
FDR and Cross-validation	0	0	0	0	1	0	2	6

* LVMass was adjusted for age of onset, sex, height, weight, hypertension

** LVEF was adjusted for age of onset, sex

*** For survival analysis, Risk Index was used to cross-validate, P-value for Cox model with Risk Index <0.1 is considered as crossvalidated. For continuous outcomes, 4-fold CV was used. Press-Rsquared >0.005 (SNP univ model) or Press-Rsquared for full model >0 and Press-Rsquared Difference between full model and submodel >0.005 (Epistasis) is considered to be crossvalidated for interaction.

The relative risks (RR) associated with these polymorphisms are listed in Table 4. The two polymorphisms with single locus effects were *ADRA2C*(-1692 T/G) and *ADRA2C*(+964/965 ins/del). However, both of these polymorphisms were involved in significant epistatic interactions making the interpretation of the relative risks of their main effects impossible. Even though we equally tested for interactions within and among the two genes, somewhat surprisingly, we only found evidence of intergenic epistasis. The interaction between *ADRA2C*(+964/965 ins/del) and *ADRB1*(+145 A/G) arises from the 10:AG genotype class having elevated risk of death or transplant with RR = 5.12 (2.66,9.88). With respect to the interaction between *ADRA2C*(-230 T/C) and *ADRB1*(-517 T/C), the TC:CT genotype class has much higher risk of death or transplant with RR = 8.31 (3.32, 20.75) compared to the reference genotype class of TT:TT. The interaction between *ADRA2C*(-230 T/C) and *ADRB1*(-2297 T/G) appears to be due to the TC:TG genotype class (RR = 9.23 (3.67,23.16)), and the interaction between *ADRA2C*(-1692 T/G) and *ADRB1*(+145 A/G) is associated with an increased risk of death or transplant in the TG:AG genotype class with RR = 4.59 (2.31, 9.12). Likewise, the interaction between *ADRA2C*(-1692 T/G) and *ADRB1*(-3641 C/T) identifies the TG:CT genotype as the high risk group with RR = 3.56 (1.80, 7.05). Finally, the *ADRA2C*(-1692 T/G) and *ADRB1*(-3255 A/C) polymorphism appear to be interacting and again the TG genotype of the *ADRA2C*(-1692 T/G) gene has a significant increase risk in a particular *ADRB1 *genotype background – namely, AC genotype – such that the TG:AC genotype class has a relative risk of 3.02(1.53,5.97).

**Table 4:** FDR Significant and Cross-Validated Epistatic Effects of *ADRA2C *and *ADRB1 *polymorphisms on survival

**SNP1**	**SNP2**	**Genotype1**	**Genotype2**	**Relative Risk**	**N**	**Model P-value**	**Cross-Validation P-value**
**Main Effects**							
***ADRA2C*(-1692 T/G)**		TT		1	544	0.029	0.031
		TG		1.34(0.91, 1.95)	74		
		GG		n/a	6		
***ADRA2C*(+964 ins/del)**		11		1	516	0.065	0.018
		10		0.51(0.07, 3.64)	65		
		00		1.58(1.07, 2.32)*	6		
**Epistatic Effects**							
***ADRA2C*(+964 ins/del)**	***ADRB1*(+145 A/G)**	00	AA	n/a	5	<0.001	0.069
		10	AA	1.20 (0.74,1.95)	39		
		10	AG	5.12 (2.66, 9.88)*	17		
		11	AA	1	349		
		11	AG	0.86 (0.59, 1.25)	108		
		11	GG	0.70 (0.31, 1.61)	15		
***ADRA2C*(-230 T/C)**	***ADRB1*(-517 T/C)**	TC	CT	8.31(3.32, 20.75)*	7	<0.001	<0.001
		TC	TT	0.46(0.15, 1.42)	16		
		TT	CC	0.51(0.21,1.24)	17		
		TT	CT	0.88 (0.62,1.26)	121		
		TT	TT	1	455		
***ADRA2C*(-230 T/C)**	***ADRB1*(-2297 T/G)**	TC	TG	9.23(3.67,23.16)*	7	0.001	0.014
		TC	TT	0.61(0.22, 1.64)	16		
		TT	GG	0.61(0.27,1.38)	18		
		TT	TG	0.99 (0.71,1.36)	137		
		TT	TT	1	422		
***ADRA2C*(-1692 T/G)**	***ADRB1*(+145 A/G)**	GG	AA	n/a	5	0.001	0.097
		TG	AA	0.99 (0.62,1.61)	44		
		TG	AG	4.59 (2.31,9.12)*	17		
		TG	GG	n/a	2		
		TT	AA	1	371		
		TT	AG	0.85(0.59, 1.22)	111		
		TT	GG	0.61(0.27, 1.38)	17		
***ADRA2C*(-1692 T/G)**	***ADRB1*(-3641 C/T)**	GG	CC	n/a	6	0.005	<0.001
		TG	CC	0.95 (0.60, 1.53)	52		
		TG	CT	3.56 (1.80,7.05)*	18		
		TT	CC	1	369		
		TT	CT	0.84 (0.58,1.21)	116		
		TT	TT	0.62 (0.27, 1.42)	17		
***ADRA2C*(-1692 T/G)**	***ADRB1*(-3255 A/C)**	GG	AA	n/a	6	0.010	<0.001
		TG	AA	1.08 (0.68,1.71)	52		
		TG	AC	3.02(1.53,5.97)*	19		
		TT	AA	1	379		
		TT	AC	0.83(0.58,1.21)	116		
		TT	CC	0.73 (0.32,1.66)	16		

* RR is significant at α < 0.05

Given some degree of non-independence among these epistatic effects, we performed an *a posteriori *pooling of all high risk two locus genotype classes with RR's whose 95% confidence interval exceed 1.0 to attempt to identify the extent of overlap between the effects from the pairs of loci. These were compared to all other genotype classes combined, which provides a way to interpret these very specific sets of polymorphisms as if being used clinically. Here we found that the combined relative risk was 3.35 (1.82, 6.18).

## Discussion

In this study, we identified multiple polymorphisms within the *ADRA2C *and *ADRB1 *genes that interact to increase risk of death or transplant in heart failure patients. We investigated these genes because numerous studies in human patient populations [20,12,14], animal models [17], and in vitro cell systems [18,11,22,23] have demonstrated their relevance to heart failure phenotypes or receptor expression/function.

As noted in the Results, four of the polymorphism significantly deviated from Hardy-Weinberg equilibrium. However, a previous study that genotyped the ADRA2C polymorphisms in a cohort of unaffected individuals did not show evidence of deviations from HWE [22]. Similarly, genotyping of the ADRB1 polymorphisms in unaffected individuals for the Seattle SNPs database  do not show significant deviations from HWE. The HW deviations we observed could be due to an underlying association with heart failure [28] since our sample is exclusively heart failure patients and these genes have been previously associated with this disease, but random chance and genotyping error cannot be excluded.

The α_2_ARs expressed on the presynaptic cardiac sympathetic nerves inhibit the release of norepinephrine when they are bound by the neurotransmitter [5], and thus provide a mechanism to regulate release of the neurotransmitter as sympathetic nervous system activity markedly increases in progressive heart failure. And indeed, in studies of gene-targeted mice where α_2_ARs have been ablated, severe cardiomyopathy results due to norepinephrine cardiotoxicity exerted through myocyte β_1_ARs [5]. The human *ADRA2C *ins/del polymorphism previously denoted Del322-325, and here denoted *ADRA2C*(+964/965 ins/del) significantly reduces the function of α_2C_AR receptors in transfected cells [18]. While Caucasians have a very low prevalence of this allele [20] it appears that other common polymorphisms within this racial group, in the promoter and 3' UTR region of the gene, affect receptor expression [22]. Of note, evidence suggests that there are few "spare receptors" in the complement of α_2C_ARs expressed in cardiac presynaptic nerves since heterozygous α_2C_AR knock-out mice (~50% less receptor) also develop cardiomyopathy under pressure overload [29]. Thus relatively small changes in expression due to polymorphisms may have physiologically relevant effects on the heart during heart failure progression. For the β_1_AR, the most frequently studied polymorphic variation is at nucleotide 1165, where Arg or Gly can be commonly found at amino acid 389. This lies within a G-protein coupling domain, and in transfected cells the Arg variant exhibits enhanced coupling to adenylyl cyclase [11]. In transgenic mice, with matched cardiac expression of the human β_1_AR Arg or Gly389 receptors, Arg hearts have enhanced contractility, but progress to failure by 9-months of age, while Gly hearts show less contractile enhancement and no pathologic effects [17]. The Arg389 phenotype also revealed a gene dose-response, and given that promoter SNPs of the *ADRB1 *gene alter expression in cell-based systems [23], the potential for cardiac relevance of non-coding β_1_AR SNPs is apparent.

The previous studies of interactions between one *ADRA2C *and one *ADRB1 *polymorphism illustrate that the physiological effects of variations in these two genes act synergistically to increase risk of heart failure [20] and influences patient responsiveness to beta-blocker therapy as assessed by short-term improvements in LVEF [21]. Our study indicates that there may be several other polymorphisms within these genes that have important physiological and clinical consequences for heart failure patients. Specifically, the *ADRA2C*(-1692 T/G) was involved in three epistatic interactions with *ADRB1 *polymorphisms (+145 A/G, -3641 C/T, and -3255 A/C) and the *ADRA2C*(-230 T/C) polymorphism was involved in two epistatic interactions with *ADRB1 *polymorphisms (-2297 T/G and -517 T/C). Only one *ADRB1 *polymorphism (+145 A/G) was involved in more than one epistatic interaction with the *ADRA2C *polymorphisms (-1692 T/G and +964/965 ins/del). In total, three polymorphisms in the *ADRA2C *gene and five polymorphisms in the *ADRB1 *gene appear to be implicated in epistatic effects on heart failure survival.

Because of the relatively large number of polymorphisms investigated in each gene one natural analytical approach might have been to investigate haplotypes within each gene and to test for potential interaction between haplotypes in their influence on risk. However, one of the main drawbacks of such an approach is reducing the haplotype space to the relevant set with phenotypic effects that could potentially interact within or across genes. Since we did not find any evidence of significant intragenic epistasis, it becomes even more difficult to analyze the diverse set of haplotypes – many with low frequency – to identify potentially relevant interactions across genes.

One of the short-comings of genetic association studies is that they have often failed to replicate and Manly [30] suggests that internal validation, common to good experimental practices, is one way to avoid the publication of spurious findings. In our study, we used cross-validation methods to significantly reduce the chance of false positives. Cross-validation methods were developed as a way to incorporate a measure of predictive accuracy (and correspondingly, a measure of prediction error) for an estimated model based on its performance predicting the outcome for independent test cases [31]. During the last decade, cross-validation methods have been used widely for everything from robust variable selection in gene expression array studies [32] to reducing false positives in gene-gene interaction studies [33,34] to evaluating the predictive accuracy of molecular or genetic classifiers of disease before clinical implementation [35]. It has become a standard in the field of metabolomic [36], proteomic [37,38], and transcriptomic [39] studies because of its ease of execution and its emphasis on prediction in independent test cases as a method of discriminating between true associations and false associations.

The epistatic interactions identified here occur between -1692, -230, and the +964/965 polymorphisms of *ADRA2C *and -3641, -3255, -2297, -517 and +145 polymorphisms in *ADRAB1*. We have previously generated constructs that mimic these polymorphisms and by transient transfection of cells ascertained expression phenotypes (5' and 3' -flanking regions) or signaling phenotypes (nonsynonymous coding polymorphisms) [11,22,23,18]. All but one of the *ADRA2C *5' promoter and 5' UTR polymorphisms found here that interact with *ADRAB1 *have been found to influence α_2C_AR expression in these model systems. (The *ADRA2C*(-1692) polymorphism has not been studied in this manner.) And, the +964/965 deletion polymorphism results in depressed agonist-promoted function [18]. For the *ADRAB1*, the aforementioned polymorphisms of the promoter region (except for -1294 which has not been studied) have also been shown to alter β_1_AR expression [23]. The nonsynonymous polymorphism at nucleotide position +145 (representing Gly49) results in a β_1_AR that undergoes enhanced agonist-promoted downregulation compared to the Ser49 receptor [40], an important phenotype since downregulation is a protective mechanism in heart failure. Taken together, then, there is biologic plausibility in the epistatic interactions that were observed. The fact that there is a relatively large fraction of the patients that do not have these specific combinations, and yet they do display variability in heart failure progression, indicates additional genetic (and likely non-genetic) causes for heterogeneity. Within the axis which we are currently exploring, there are a number of other genes to be considered. As additional information from fine mapping and cell-based studies is obtained, there is the possibility for enhanced predictive power and an assignment of risk alleles for a greater percentage of heart failure patients. Nevertheless, the current work shows that epistatic interactions between α_2C_AR and β_1_AR polymorphisms affect heart failure survival, and further confirm the notion that this complex syndrome is modified by multiple polymorphisms in multiple genes.

## Conclusion

Although we did not observe intragenic epistatic interactions between the *ADRA2C *and *ADRB1 *genes, we did observe multiple polymorphisms acting synergistically between the *ADRA2C *and *ADRB1 *genes to increase risk of death or cardiac transplant in heart failure patients. This underscores the complexity of the genetic factors that affect the progression of this syndrome.

## Competing interests

The authors declare that they have no competing interests.

## Authors' contributions

SLRK performed statistical analysis and drafted the manuscript. RK performed statistical analysis and assisted in drafting the manuscript. MK, BA, and GG developed genotyping methods for the *ADRA2C *and *ADRB1 *polymorphisms and performed the genotyping of the subjects. HH and LW recruited subjects and interpreted echocardiogram results. KC managed the subject clinical and endpoint data. GWD designed the study and assisted in drafting the manuscript. SL identified the *ADRA2C *and *ADRB1 *polymorphisms and their haplotypes, developed genotyping methods, participated in the design of the study and interpretation of results, and assisted in drafting the manuscript. All authors read and approved the final manuscript.

## Pre-publication history

The pre-publication history for this paper can be accessed here:


